# The motivations and practices of vegetarian and vegan Saudis

**DOI:** 10.1038/s41598-023-36980-x

**Published:** 2023-06-16

**Authors:** Aroub Alnasser, Norah Alomran

**Affiliations:** Food Science and Nutrition Department, College of Food and Agriculture Sciences, King Saud University, 11495 Riyadh, Saudi Arabia

**Keywords:** Psychology and behaviour, Sustainability

## Abstract

In the Middle East, particularly in Saudi Arabia, the offering of a meat dish to guests is a deeply embedded cultural tradition, and a meat-based diet is the standard in Saudi Arabia. Thus, the rise of veganism and vegetarianism within Saudi Arabia is surprising and worthy of attention, as is understanding the perceptions and motivations behind this phenomenon, particularly as they relate to food and sustainability. This research was designed to investigate this emerging phenomenon and to identify key differences in dietarian identity between Saudi vegetarians and vegans using Rosenfeld and Burrow's Dietarian Identity Questionnaire. Among other results, the vegan group scored significantly higher on the prosocial motivation construct, suggesting the desire to help society as a whole is a stronger motivating factor for vegans. As well, the vegan cohort scored higher in the personal motivation category. From an environmental and public health perspective, understanding the key factors motivating individuals to adopt a vegetarian or vegan diet in a meat-based culture like Saudi Arabia can be used to encourage others to pursue more healthy and sustainable food behaviors.

The World Bank^1^ recognizes “social sustainability” as the effort “to foster more resilient and peaceful communities.” Food culture, availability, and behavior play an integral role in this process. Hospitality is an ingrained sociocultural practice in the Middle East, particularly in Saudi Arabia, and is “synonymous with the Arab world because Arabs are ‘famed’ for the hospitality they show to their guests”^2^^(p1)^. Traditionally, the offering of a meat dish to guests is a deeply embedded cultural tradition within the Saudi community^3^. Moreover, a carnivorous or meat-based diet is the standard diet in Saudi Arabia^4^. Thus, the rise of plant-based diets is surprising and worthy of attention. A recent cross-sectional study conducted in Saudi Arabia found a significant rise of interest in the dietary practices of vegetarians and vegans, concluding that “vegetarianism appears to be a growing phenomenon among the Saudi population.”^5^ ^(p. 1)^.

Historically, the traditional Saudi diet incorporates moderate to high levels of meat consumption^6^. Due to rapid economic growth and a transition towards a Westernized lifestyle, other meat products and fast-food meat options are becoming increasingly popular throughout the country^7^. Given the intertwined nature of diet and culture, a vegetarian or vegan diet is considered a nonstandard dietary identity when it is not the predominant dietary choice within a culture or family^8^. Understanding why more individuals are now making this shift to a non-conforming diet is paramount to furthering our understanding of modern Saudi society in flux and the cultural, health, and societal issues Saudis must navigate. Within Saudi Arabia specifically, this shift may reflect changing societal attitudes—on sustainability, health, or ethics–for example, or may be unique to each individual, with no detectable cultural shift.

The rates of vegetarianism and veganism are rapidly increasing in many countries worldwide for a complex and diverse array of reasons^7,9,10^. Recent research amongst Saudis suggests that the appearance of vegetarian and vegan diets has substantially increased throughout Saudi Arabia in recent years (13%)^11,12^. However, little to no data exists to explain the uptick in popularity, the demographics of the individuals converting to vegetarianism and veganism, or the motivations behind their dietary lifestyle changes.

Vegetarianism and veganism are two dietitian identities that have seen rapid growth in recent years. The motivations for being/becoming vegetarian or vegan are diverse and can be seen not only as a dietary identity but also as a cultural identity^13^ and influenced by social movements based on the perceived benefits of a vegetarian/vegan diet^14^. Vegetarianism in the Western world has been found to be primarily motivated by animal welfare concerns with health considerations being second and environmental sustainability third^15^. This differs from Saudi Arabia where a recent study identified the leading factors for choosing a vegetarian diet to be the perceived health benefits, followed by care for animals and environmental concern^5^.

Globally, the plant-based meat market is expected to grow by 15.8% between 2020 and 2027^16^. Furthermore, it has been reported within other population groups, for example, that meat consumption may fall at a faster rate than the number of individuals identified as vegetarian is rising^17^. This suggests that non-meat eaters do not necessarily identify as vegetarians and that occasional and low-meat eaters may relate to vegetarian or vegan ideals^18,19^. The results also point to the intricacies of dietarian identification and dietary practices; people may not identify as vegetarian or align with and practice perceived vegetarian ideals, despite consuming a solely plant-based diet^20^. Research conducted by Kirsten et al.^21^ demonstrated that people regularly self-categorize as a type of dieter that does not correspond to the definition of that diet, for example, 50% of pescatarians self-identified as vegan or vegetarians. Interestingly, non-vegan or vegetarian consumers are also shifting their food habits towards vegan food options^22^. Research suggests that this is primarily driven by concerns for animal welfare, the environment, and the health of the individual^23,24^.

Although health-related factors are responsible for some individuals adopting vegetarian or vegan diets^25^, there are other driving factors for a separate subset of vegetarian individuals. There is a growing body of evidence outlining the significant impact that our dietary choices have on the environment, this data has become increasingly important as the global population levels (and therefore food production) increase dramatically^26^. Agriculture for meat production produces considerable greenhouse gas emissions, which are a leading contributor to global warming and climate change^27^. This is one reason why vegan and vegetarian diets are considered a more sustainable alternative with a smaller ecological footprint than carnivorous or omnivorous diets^28,29^ and a potentially important mitigation strategy against climate change^30^.

Indeed, previous research has suggested that individuals pursuing a vegan/vegetarian diet more frequently purchase second-hand or ecologically friendly products compared to their omnivorous counterparts^31^. These differences suggest that for many individuals, choosing a specific dietary pattern represents a greater overall decision than simply the foods they prefer to eat. Food and culture have been intertwined for millennia, and the consumption of meals has been considered a social event for many centuries. For many individuals, the decision to eat (or not eat) a specific food can represent a greater desire to influence our own health or the health of the planet^32,33^.

Although it is likely that environmental sustainability, along with health factors and many other reasons, plays a role in motivating individuals to adopt a plant-based diet, there is currently limited data available in Saudi Arabia that quantify the motives for such dietary choices^34^.

One tool which has proven to be highly effective in providing a quantitative analysis of the dietarian identity variables of a set of individuals is Rosenfeld and Burrow's^35^ Dietarian Identity Questionnaire (DIQ). The DIQ assesses how individuals think, feel, and behave when eating or not eating animal products. The DIQ has been validated for use specifically for questions related to vegetarianism^36^ and provides valuable insights into the motives of such individuals and their dietary choices. The DIQ both understands and proves the heterogeneity of vegetarianism: the moral and ethical values of those within this subset of the population vary as much as the foods on their plate.

To provide this quantitative analysis, the DIQ uses questions tailored to provide details regarding an individual’s identity evaluations towards their diet. As a tool, the DIQ can elucidate the way an individual feels about themselves, as well as others, when it comes to dietary choices. One of the key features of the DIQ is the ability to offer a distinction between the outgroup and the ingroup. Made up of 33 items, the DIQ can be used to define eight factors surrounding dietary identity—centrality, private regard, public regard, outgroup regard, prosocial motivation, personal motivation, moral motivation, and strictness.

The centrality factor represents one’s self-concept of food. Private regard offers insight relating to the positive evaluation of the ingroup; dietary “peers” who follow the same eating pattern as the individual. Conversely, public regard is used to identify the degree of perceived negativity from the outgroup. Outgroup regard refers to the individual´s evaluation of those with an eating pattern that’s different to their own. The motivation factors represent justification for why an individual pursues a given eating pattern. For example, Prosocial motivation represents how motivated an individual is by the thought of their diet benefiting others. Personal motivation is the motivation of pursuing a dietary pattern because it benefits one’s own health and wellbeing. Moral motivation refers to personal morality as a motivating factor for pursuing a given dietary pattern. Strictness refers to how rigidly one holds themselves to their dietary patterns.

The definition of each of the eight factors is based on proposed ideas and models, such as the united model of vegetarian identity (UMVI), by Rosenfeld & Burrow^37^. Rosenfeld^36^ previously used the DIQ to examine the dietary identity differences between vegan and vegetarian groups. According to their findings, vegans follow their dietary patterns more rigidly compared to their vegetarian counterparts. In addition, vegans have a more negative opinion of the outgroup, a more positive opinion of their ingroup, and have a higher level of centrality (suggesting that their diet is more intertwined with their sense of self) compared to vegetarians.

This study aims to build on previous data while offering insights into the dietary motivations of a specific population. By identifying the motives of Saudis who have chosen to follow a vegetarian or vegan diet, this research provides insight into dietarian perceptions and identities in Saudi Arabia. The primary objective of this study was to use the DIQ to identify eight key differences between two dietary subsets.

This study also hopes to not only focus on the growth of the subsets of veganism and vegetarianism but to also address the dearth of extant literature and research that focuses on Rosenfeld’s DIQ to examine dietary motivations and practices among Saudis. Our findings shed new light on the impact of the individuated Saudi dietarian identities of veganism and vegetarianism. The study’s findings offer unique insights into the practices and driving factors—including environmental or ethical consciousness—behind the beliefs, attitudes, and dietary preferences among Saudi vegans and vegetarians.

## Methods

This groundbreaking research was designed to address the limited literature on dietarian identity, specifically veganism and vegetarianism^38^ in Saudi Arabia. Although the Saudi Arabian population's adoption of vegetarianism/vegetarian diets has not been fully investigated, global survey findings in 63 countries, including Saudi Arabia, indicated that 16% of the Africa/Middle East region are vegetarian^39^. In addition, a recent study examining the effect of dietary weight management programs on health parameters within a Saudi university community^40^ suggested that approximately 16% of the participants were vegetarian. If we assume that 16% (2,016,000) of Saudi Arabia’s current population of 12.6 million adults aged ≥ 18 years^41^ are vegetarian, and we use the Raosoft sample size online calculator with 50% response distribution, 5% margin of error and 90% confidence level, the minimum recommended sample size was 271. The researchers employed a virtual snowball sampling approach to identify hidden populations^42^.

Snowball sampling was used to provide researchers with access to vegetarian and vegan adherents in Saudi Arabia. To implement a snowball sampling strategy, the web-based survey link was posted on social media networks (WhatsApp and Telegram) for 4 weeks in spring 2021 to publicize the survey and connect with participants. The participants were also encouraged to share links with their social networks. A total of 338 volunteers were recruited, with a final recruitment of 330 participants. Of the 338 volunteers, eight (2.4%) responses were excluded as they either did not agree to participate (n = 2) or they did not meet the inclusion criteria (n = 6). The inclusion criteria were as follows: (1) aged at least 18 years, (2) both men and women, (3) practicing a vegan or vegetarian diet, and (4) Saudi Arabia. The exclusion criteria were as follows: (1) age < 18 years and (2) participants who did not practice a vegan or vegetarian diet. For this survey, no personal information was collected, and participant anonymity was ensured and protected. Informed consent was obtained from all the participants.

### Measures

The material foundation of this study included Rosenfeld and Burrow’s^35^ DIQ model of dietarian identity as validated by Rosenfeld^36^. Information gathered from the participants was used to investigate whether vegetarians and vegans think, feel, and behave divergently with respect to their diets^20^. Dietarian identity expands beyond dietary patterns and self-labeling and comprises eight psychological variables^35^; see Table 1 for variables and conceptual definitions. Responses to all the items ranged from 1 (strongly disagree) to 7 (strongly agree).

**Table 1:** Conceptual definitions of DIQ variables, (adapted from Rosenfeld 20).

Definition	Concept
Centrality	One’s views of a dietary pattern is a predominant feature of one’s self-concept
Private regard	Personal feelings toward following a dietary pattern and toward others who also eat this way
Public regard	Feelings about how members of dietary out-group members, and society at large, evaluate those who follow one’s dietary pattern
Out-group regard	One’s evaluation of people who follow a dietary pattern that differs from one’s own
Prosocial motivation	A desire to help others and society as a whole is a reason for following one’s dietary pattern
Personal motivation	A desire to help oneself as a reason for following one’s dietary pattern
Moral motivation	Values and beliefs about ethics and principles as a reason for following one’s dietary pattern
Strictness	One’s consistency and adherence to a dietary pattern

The questionnaire was professionally translated from English into Arabic. After translation, to ensure the translated content validity, two health professionals at King Saud University, reviewed the questionnaire. Minor revisions were made based on their recommendations. The face validity of the questionnaire was determined using a small sample of Saudi adults (n = 10) and revised based on their feedback. During this process, Cronbach's alpha was used to determine the internal consistency reliability. An adequate value of 0.63 was obtained. The final version of the Web-based questionnaire was posted on Google Forms.

### Procedure

Informed consent was obtained from participants prior to completing the survey. Once participants clicked on the link to the study, the title and purpose of the study were displayed to the participants. To proceed, the participants were required to read and accept the study's purpose. If they declined, they were not allowed to continue the survey. Consenting participants provided demographic information (age, sex, residency, education/employment, weight, height, and presence of chronic disease) prior to survey completion.

The survey followed ethical research practices and was performed in accordance with relevant guidelines and regulations. The study was reported in accordance with the Consensus-Based Checklist for Reporting of Survey Studies (CROSS) guideline^43^. The study was conducted in accordance with the Declaration of Helsinki and approved by the Scientific Research Ethics Committee at King Saud University (Ref No: HE-21-191).

The data obtained were analyzed using the Statistical Package for the Social Sciences (SPSS) system (Version 22.0). Descriptive statistics (frequency, percentages, and standard error), chi-square test, and correlation test were applied, as appropriate. Statistical significance (*P* < 0.05) is indicated as appropriate. Confidence intervals (CI) were reported as 93% for each analysis.

## Results

### Demographic data

The socio-demographic data (Table 2) details the personal characteristics of the 330 study participants who responded by dietary type. The factors evaluated include age, education level, BMI, gender, employment status, and the presence of chronic disease.

**Table 2:** Personal characteristics of the study subjects by type of diet.

Variables	Vegetarian diet	Vegan diet	Total	*P* value
Age (years) (mean ± SD)	22.7 ± 6	22.4 ± 5	22.5 ± 5.6	0.597
BMI (kg/m^2^) (mean ± SD)	23.2 ± 5	22.6 ± 6	22.8 ± 6	0.386
Gender				0.068
Female	125 (37.9%)	176 (53.3%)	301 (91.2%)	
Male	7 (2.1%)	22 (6.7%)	29 (8.8%)	
Education level				0.003*
Primary	0 (0.0%)	1 (0.3%)	1 (0.3%)	
High school	27 (8.2%)	64 (19.4%)	91 (27.6%)	
Diploma	5 (1.5%)	10 (3.0%)	15 (4.5%)	
Bachelor's degree	82 (24.8%)	116 (35.2%)	198 (60.0%)	
Postgraduate	18 (5.5%)	7 (2.1%)	25 (7.6%)	
Employment				0.368
Student	96 (29.1%)	136 (41.2%)	232 (70.3%)	
Employee	19 (5.8%)	22 (6.6%)	41 (12.4%)	
Free work	6 (1.8%)	11 (3.3%)	17 (5.2%)	
Not working	11 (3.3%)	27 (8.2%)	38 (11.5%)	
Retired	0 (0.0%)	2 (0.6%)	2 (0.6%)	
Presence of chronic disease				0.009*
No	77 (58.3%)	143 (72.2%)	220 (66.7%)	
Yes	55 (41.7%)	55 (27.8%)	110 (33.3%)	
BMI				0.072
Underweight	20 (6.1%)	34 (10.3%)	54 (16.4%)	
Normal weight	75 (22.7%)	131 (39.7%)	206 (62.4)	
Overweight	22 (6.7%)	16 (4.8%)	38 (9.7)	
Obesity	15 (4.5)	17 (5.2%)	2 (9.7%)	
Total	132 (40.0%)	198 (60.0%)	330 (100%)	

*P* < 0.05: Significant, * statistically significant.

When vegetarian participants were compared to their vegan counterparts (Table 2), statistically significant differences presented themselves in terms of the education level of the two groups (*P* = 0.003). While vegans were more likely to have graduated from high school and undergraduate school, vegetarians were more likely to have attended postgraduate school. In the presence of chronic disease (*P* = 0.009), considerably more vegetarians (71.4%) suffered from chronic disease as compared to (38.5%) of those following a vegan diet. No significant differences were identified between the two groups in terms of age, BMI, or employment status. Although more participants were female (301 versus 29 male participants), the gender disparity was equally divided between the vegetarian and vegan groups.

### DIQ variables between Vegetarians and Vegans

Next, the survey results were analyzed, and differences were identified between vegetarian and vegan cohort members with regard to the eight variables of the DIQ (Table 3).

**Table 3:** DIQ differences between vegetarians and vegans.

DIQ definitions/type of diet (*P* value)	N	Mean ± STD
Centrality (*P* = 0.007*)		
Vegetarian	132	4.8 ± 1.5
Vegan	198	5.3 ± 1.6
Private regard (*P* = 0.087)		
Vegetarian	132	5.0 ± 1.4
Vegan	198	5.3 ± 1.5
Public regard (*P* = 0.833)		
Vegetarian	132	2.6 ± 1.3
Vegan	198	2.6 ± 1.5
Out-group regard (*P* = 0.006*)		
Vegetarian	132	5.7 ± 1.3
Vegan	198	5.3 ± 1.4
Prosocial motivation (*P* < 0.001**)		
Vegetarian	132	4.4 ± 1.7
Vegan	198	5.2 ± 1.5
Personal motivation (*P* = 0.001*)		
Vegetarian	132	5.3 ± 1.7
Vegan	198	5.9 ± 1.5
Moral motivation (*P* < 0.001**)		
Vegetarian	132	4.4 ± 1.9
Vegan	198	5.4 ± 1.9
Strictness (*P* = 0.425)		
Vegetarian	132	5.6 ± 1.8
Vegan	198	5.7 ± 1.5

*P* < 0.05: Significant, * statistically significant, ** highly statistically significant.

The results for each individual variable are shown in (Supplementary Material). Those following a vegan diet scored higher in centrality, lower in out-group regard, higher in prosocial motivation, higher in personal motivation, and higher in moral motivation. All the differences were statistically significant (*P* < 0.05).

Although no statistically significant differences were identified between vegetarians and vegans in terms of private regard, the vegan cohort scored higher in each of the three private-regard items, suggesting a potential trend, although this was not observed for public-regard items. Similarly, no trend was observed for strictness items (see Supplementary Materials for details).

## Discussion

The results of this study indicate that there are differences between vegetarians and vegans in terms of eight dietarian identity constructs. This study’s findings align with all but two of Rosenfeld’s^20^ discoveries that revealed that “Vegans saw their dietary patterns as more intertwined with their identity (higher centrality).” This present research found that in terms of centrality, those identifying as vegans scored significantly higher than their vegetarian counterparts, suggesting that vegans feel more strongly that their diet makes up an important part of their self-identity. In terms of prosocial motivation and out-group regard, this study’s findings also agree with Rosenfeld’s results which found that vegans had a stronger foundation of motivations to practice their dietary patterns (higher prosocial, personal, and moral motivations). As with Rosenfeld’s^20^ study, this research also found no significant difference between the groups in the category of “strictness.” However, unlike Rosenfeld who “found support for all seven of my [his] hypotheses, with vegetarians and vegans differing from one another along all dietarian identity dimensions except for strictness,”^20^ ^(p. 14)^ this study did not find significant differences between vegans and vegetarians in terms of private and public regard. Differences in this and Rosenfeld study results may speak to the disparate cultural, religious, and geographical contexts of the respective studies.

As noted, in terms of centrality, those identifying as vegans scored significantly higher than their vegetarian counterparts, suggesting that vegans feel more strongly that their diet makes up an important part of their self-identity. This is a somewhat intuitive result: those who follow a vegan diet face fewer options when eating socially, either in private gatherings or restaurants. Therefore, it could be extrapolated that vegans are forced to make more decisions that relate directly to their diet in terms of where, how, and what to eat, which in turn causes a greater sense of centrality to their personal identity. This is in agreement with Reuber & Muschalla's study^44^, conducted in Germany, which found that vegans experience their dietary practices as more central to their identity than vegetarians. Vegetarians and vegans also differed in out-group regard, with vegans exhibiting a significantly lower out-group regard. Interestingly, another study conducted in Germany found that vegans reported more extreme scores in the Rosenfeld questionnaire than vegetarians^21^. This suggests that vegans have a lower opinion of individuals who follow a diet different from their own.

In terms of the factors that motivate individuals to pursue their dietary choices (prosocial, personal, and moral motivations), significant differences were observed between the vegetarian and vegan groups. The vegan group scored significantly higher on the prosocial motivation construct, suggesting that the desire to help society as a whole is a stronger motivating factor for vegans than vegetarians when it comes to their dietary decisions. These results are similar to those found in other studies such as Reuber & Muschalla's study^44^ which found a correlation between heightened moral motivation and the centrality of dietary identity. In this study the vegan cohort scored higher in the personal motivation category, indicating that individuals following a vegan diet may seek to improve their health outcomes more than their vegetarian counterparts do.

No differences were identified between the two groups in either the private or public regard category, suggesting that vegans and vegetarians held similar views of those who shared their dietary choices as well as similar views regarding how they may also feel more persecuted or stigmatized by those who do not share their dietary views. Additionally, no difference was identified between the vegans and vegetarians in terms of strictness in terms of dietary adherence. While many perceived veganism as a stricter dietary discipline than vegetarianism, this shows that the two groups adhered to their dietary choices with equal tenacity. These results differ from a Kirsten study^21^ which found that the more animal products participants removed from their diet the higher the centrality of dietary identity is and therefore the higher the strictness of adherence to one's diet.

Although the sample size (n = 330) for this study was relatively small, the internal validity of each component was strengthened by the fact that there was limited variation between each result within a category. In this sense, these data are consistent with findings previously reported using the DIQ^9,35^. A similar study^45^ conducted in the USA compared the dietarian identity profiles of a cross-section of 992 vegetarian and vegan subjects. Although not of a specific race, culture, or ethnicity, this study used almost identical methods and identified significant differences between vegetarians and vegans in seven of the eight dietarian identity constructs. In addition to the five differences identified in this study, the Rosenfeld study^46^ also identified differences in both private and public regards. In both studies, no differences in strictness constructs were identified.

In the Rosenfeld study^36^, vegans had significantly higher private regard and lower public regard than vegetarians, suggesting that those adopting a vegan diet may view their peers in a more favorable light and may also feel more persecuted or stigmatized by others who do not share their dietary views. The fact that Rosenfeld’s results were not replicated in this study (with no significant difference identified between the two groups for these categories) may indicate that vegans and vegetarians within the Saudi population identify differently.

Based on the data obtained from this survey, Saudi vegetarians and vegans perceive those sharing their dietary views similarly, and vegans do not feel more stigmatized or cast out by non-vegans. One explanation for this result could be that the majority of Saudi vegans and vegetarians adopt these diets due to health reasons, rather than moral or ethical motivators as demonstrated by a recent study on vegetarianism in Saudi Arabia^5^. Those adopting a vegetarian or vegan diet for health reasons may be less likely to relate to other elements often associated with vegetarian and vegan lifestyles, such as activism^47^ or strong political views^48^. Compared to those making dietary choices based on the pursuit of a healthy lifestyle, political motives can lead to animosity and a greater inability to relate to those who do not share a given dietary view^49^.

In addition, various studies have indicated that young Saudi women and adolescents are searching for weight loss solutions^33,34^, which could be a driving factor for some individuals to turn to vegetarian or vegan diets. The most recent meta-analysis research consisting of 436,178 people^50^ with various dietary identities found a significant positive relationship between plant-based diets and body composition, suggesting that vegetarian and vegan diets are associated with the pursuit of weight loss. As the mean age of participants in this study was 22 years, and a vast majority (91%) were female, weight management and body image could be important driving factors that are encouraging young women to pursue a vegetarian or vegan diet.

In terms of study limitations, the fact that the study’s respondents were primarily young women with high personal motivations may partially explain the differences between these findings and those of the Rosenfeld study^46^. Moreover, this study was a cross-sectional data analysis, whereas a longitudinal study could shed more light on the evolution of the eight segments in the DIQ method. In addition, the study should have been validated after translation. As well, the survey was limited to single-mode data collection and only accessible online. Participants without internet access could not participate. To guarantee the safety protocols during the COVID-19 pandemic, online survey recruitment, completion, and collection were performed, and no participants were interviewed in person. In comparing vegans and vegetarians, there was no attempt to address the influence of BMI, gender, education, etc. on the DIQ scores. Also, this study did not include an omnivorous reference group. The scope of the survey and study was limited to the differences between two dietary subsets as measured and considered using the framework of eight key variables within the DIQ.

Despite these limitations, this study not only provides further support for the use of the DIQ as a measure of vegetarian and vegan motives but also offers a novel insight into the use of the DIQ to explore dietary identification within a specific cultural or geographical subpopulation. Indeed, identifying the motives of individuals within a specific population can provide local communities with insights into how to better serve the dietary needs of their constituents. Interestingly, research has suggested that local food communities not only play an important role in offering individuals locally sourced produce and food but also strengthen an individual’s food-related self-image^51^.

Additionally, understanding the key factors motivating individuals to adopt vegetarian or vegan diets is of great importance at different levels. The attitudes, beliefs, and behaviors of those who adopt plant-based diets can provide more perspective for future research and interventions. From an environmental perspective, these findings can be used to encourage others to eat more sustainably. However, in terms of the direction and thrust of ongoing and upcoming research, it is hoped that the scope of future research will achieve a greater balance in regards to geographical focus. In a review of the extant literature of studies on veganism and vegetarianism, Salehi et al.,^19^ discovered “a geographical dominance” and noted that research interest “is particularly robust within English-speaking Western countries.”^19^ ^(p. 16)^ They further describe “a geographical gap” and identify the breakdown of the studies reviewed as mainly focused on the US and the UK and, primarily “focused on developed countries, mainly in the US (33%), the UK (10%), Germany (6.5%), Australia (3.5%), Canada (3.3%), and Spain (3.3%).”^19^ ^(p. 5)^ Even though vegetarianism, as a practice and identity, traces back to ancient Greece, the presence of studies on vegetarianism and veganism is asymmetrical, favoring studies in the West. As Ruby^10^ notes of his research “the review highlights the extremely limited cultural scope of the present data and calls for a broader investigation across non-Western cultures.” This research hopes to answer that call.

## Conclusion

The DIQ is a valuable tool for deeper and more contextual understanding of dietary identities and behaviors. Understanding the key factors motivating individuals to adopt a vegetarian or vegan diet is of great personal and public health importance. Dietary motivating factors can be optimized by health practitioners and public health professionals confronted by the high prevalence of heart disease and hypertension and an obese and overweight epidemic to encourage individuals to consider the benefits of reduced meat consumption.

Understanding the key factors motivating individuals in a meat-based culture like Saudi Arabia to adopt a vegetarian or vegan diet is also of great importance in terms of social sustainability. These findings can be used to optimize and encourage others to consider more sustainable plant-based diets that are becoming increasingly associated with sustainable and resilient societies.

## Supplementary Information

Supplementary Tables.

## Data Availability

The data is not publicly available due to privacy and ethical restrictions. The data presented in this study are available on request from the corresponding author.
