# Supplementary material for: The motivations and practices of vegetarian and vegan Saudis
Source: Sci Rep. 2023 Jun 16;13:9742. doi: 10.1038/s41598-023-36980-x (PMC10275927; doi:10.1038/s41598-023-36980-x)
Supplement: Supplementary file 1 — Supplementary Tables. [file 41598_2023_36980_MOESM1_ESM.docx]

**Supplementary Materials**

**Table S1. Differences between study subjects according to Centrality items, as assessed on scales ranging from 1 to 7.**

| **Item / Diet type (p-value)** | **N** | **Mean** | **Description** |
| --- | --- | --- | --- |
| My dietary pattern is an important part of how I would describe myself. (p=0.002*) |  |  |  |
| Vegetarian | 132 | 4.5±1.8 | SoA |
| Vegan | 198 | 5.2±1.8 | SoA |
| My dietary pattern has a big impact on how I think of myself. (p=0.219) |  |  |  |
| Vegetarian | 132 | 5.0±1.9 | SoA |
| Vegan | 198 | 5.2±2.0 | SoA |
| A big part of my lifestyle revolves around my dietary pattern. (p<0.001**) |  |  |  |
| Vegetarian | 132 | 4.9±1.8 | SoA |
| Vegan | 198 | 5.6±1.6 | A |
| My dietary pattern defines a significant aspect of who I am. (p=0.073) |  |  |  |
| Vegetarian | 132 | 4.8±1.7 | SoA |
| Vegan | 198 | 5.2±1.9 | SoA |
| Following my dietary pattern is an important part of who I am. (p=0.066) |  |  |  |
| Vegetarian | 132 | 4.9±1.8 | SoA |
| Vegan | 198 | 5.3±1.8 | SoA |
| **Centrality definition total. (p=0.007*)** |  |  |  |
| Vegetarian | 132 | 4.8±1.5 | SoA |
| Vegan | 198 | 5.3±1.6 | SoA |

**SD, strongly disagree; D, disagree; SoD, somewhat disagree; Un, undecided; SoA, somewhat agree; A, agree; SA, strongly agree.**

**Table S2.** Differences between study subjects according to Out-Group Regard, as assessed on scales ranging

from 1 to 7

| **Item / Diet type (p-value)** | **N** | **Mean** | **Description** |
| --- | --- | --- | --- |
| I view people as less moral for eating foods that go against my dietary pattern. (p<0.001**) |  |  |  |
| Vegetarian | 132 | 5.6±1.5 | A |
| Vegan | 198 | 4.8±1.9 | SoA |
| I judge people negatively for eating foods that go against my dietary pattern. (p=0.021*) |  |  |  |
| Vegetarian | 132 | 6.0±1.4 | A |
| Vegan | 198 | 5.6±1.6 | A |
| Seeing people eat foods that go against my dietary pattern makes me upset or angry. (p=0.225) |  |  |  |
| Vegetarian | 132 | 5.7±1.6 | A |
| Vegan | 198 | 5.5±1.7 | A |
| If I see someone eat foods that go against my dietary pattern, I like him or her less. (p=0.051) |  |  |  |
| Vegetarian | 132 | 5.9±1.5 | A |
| Vegan | 198 | 5.6±1.8 | A |
| It bothers me when people eat foods that go against my dietary pattern. (p=0.072) |  |  |  |
| Vegetarian | 132 | 5.8±1.6 | A |
| Vegan | 198 | 5.4±1.6 | A |
| Seeing someone eat foods that go against my dietary pattern makes him or her less attractive to me. (p=0.065) |  |  |  |
| Vegetarian | 132 | 5.5±1.8 | A |
| Vegan | 198 | 5.1±1.9 | SoA |
| People should feel guilty about eating foods that go against my dietary pattern. (p=0.009*) |  |  |  |
| Vegetarian | 132 | 5.5±1.8 | A |
| Vegan | 198 | 4.9±2.1 | SoA |
| **Outgroup regard definition total. (p=0.006*)** |  |  |  |
| Vegetarian | 132 | 5.7±1.3 | A |
| Vegan | 198 | 5.3±1.5 | SoA |

**SD, strongly disagree; D, disagree; SoD, somewhat disagree; Un, undecided; SoA, somewhat agree; A, agree; SA, strongly agree.**

**Table S3.** Differences between study subjects according to Prosocial Motivation, as assessed on scales ranging from 1 to 7.

| **Item / Diet type (p-value)** | **N** | **Mean** | **Description** |
| --- | --- | --- | --- |
| I view my dietary pattern as a way of making the world a better place for others. (p<0.001**) |  |  |  |
| Vegetarian | 132 | 5.1±2.0 | SoA |
| Vegan | 198 | 5.9±1.6 | A |
| Concerns about social issues motivate me to follow my dietary. (p=0.021*) |  |  |  |
| Vegetarian | 132 | 3.1±1.9 | SoD |
| Vegan | 198 | 3.6±2.0 | Un |
| I follow my dietary pattern because I want to benefit society. (p=0.001*) |  |  |  |
| Vegetarian | 132 | 4.1±2.0 | Un |
| Vegan | 198 | 4.9±2.0 | SoA |
| I feel motivated to follow my dietary pattern because I am concerned about the effects of my food choices on other beings. (p=0.002*) |  |  |  |
| Vegetarian | 132 | 4.9±2.2 | SoA |
| Vegan | 198 | 5.7±2.0 | A |
| I am motivated to follow my dietary pattern because I want to help others. (p<0.001**) |  |  |  |
| Vegetarian | 132 | 4.2±2.1 | Un |
| Vegan | 198 | 5.3±2.0 | SoA |
| I follow my dietary pattern because eating this way is good for the world. (p<0.001**) |  |  |  |
| Vegetarian | 132 | 5.0±2.1 | SoA |
| Vegan | 198 | 5.8±1.7 | A |
| **Moral motivation definition total. (p<0.001**)** |  |  |  |
| Vegetarian | 132 | 4.4±1.7 | Un |
| Vegan | 198 | 5.2±1.5 | SoA |

**SD, strongly disagree; D, disagree; SoD, somewhat disagree; Un, undecided; SoA, somewhat agree; A, agree; SA, strongly agree.**

**Table S4.** Differences between study subjects according to Personal Motivation, as assessed on scales ranging from 1 to 7.

| **Item / Diet type (p-value)** | **N** | **Mean** | **Description** |
| --- | --- | --- | --- |
| I follow my dietary pattern because I am concerned about the effects of my food choices on my own well-being. (p<0.001**) |  |  |  |
| Vegetarian | 132 | 5.0±2.1 | SoA |
| Vegan | 198 | 5.8±1.7 | A |
| I follow my dietary pattern because eating this way improves my life. (p=0.001*) |  |  |  |
| Vegetarian | 132 | 5.7±1.7 | A |
| Vegan | 198 | 6.2±1.4 | SA |
| When thinking about which animal products to consume, I consider the effects of my food choices on my own health. (p=0.027*) |  |  |  |
| Vegetarian | 132 | 5.2±2.0 | SoA |
| Vegan | 198 | 5.7±1.9 | A |
| **Personal Motivation definition total. (p=0.001*)** |  |  |  |
| Vegetarian | 132 | 5.3±1.7 | SoA |
| Vegan | 198 | 5.9±1.5 | A |

**SD, strongly disagree; D, disagree; SoD, somewhat disagree; Un, undecided; SoA, somewhat agree; A, agree; SA, strongly agree.**

**Table S5.** Differences between study subjects according to Moral Motivation, as assessed on scales ranging from 1 to 7.

| **Item / Diet type (p-value)** | **N** | **Mean** | **Description** |
| --- | --- | --- | --- |
| I feel that I have a moral obligation to follow my dietary pattern. (<0.001**) |  |  |  |
| Vegetarian | 132 | 4.7±2.1 | SoA |
| Vegan | 198 | 5.6±1.9 | A |
| I am motivated to follow my dietary pattern because eating foods that go against my dietary pattern is immoral. (<0.001**) |  |  |  |
| Vegetarian | 132 | 4.1±2.1 | Un |
| Vegan | 198 | 5.1±2.1 | SoA |
| I follow my dietary pattern because eating this way is the morally right thing to do. (<0.001**) |  |  |  |
| Vegetarian | 132 | 4.6±2.0 | SoA |
| Vegan | 198 | 5.5±2.0 | A |
| **Moral Motivation definition total.** (<0.001**) |  |  |  |
| Vegetarian | 132 | 4.4±1.9 | SoD |
| Vegan | 198 | 5.4±1.9 | A |

**SD, strongly disagree; D, disagree; SoD, somewhat disagree; Un, undecided; SoA, somewhat agree; A, agree; SA, strongly agree.**

**Table S6.** Differences between study subjects as it relates to Private Regard and as assessed on a scale ranging from 1 to 7

| **Item / Diet type (p-value)** | **N** | **Mean** | **Description** |
| --- | --- | --- | --- |
| People who follow my dietary pattern tend to be good people. (p=0.193) |  |  |  |
| Vegetarian | 132 | 4.2±1.8 | Un |
| Vegan | 198 | 4.4±1.9 | SoA |
| Following my dietary pattern is a respectable way of living. (p=0.081) |  |  |  |
| Vegetarian | 132 | 5.4±1.7 | A |
| Vegan | 198 | 5.7±1.6 | A |
| People who follow my dietary pattern should take pride in their food choices (p=0.168) |  |  |  |
| Vegetarian | 132 | 5.4±1.7 | A |
| Vegan | 198 | 5.7±1.7 | A |
| **Private regard definition total. (p=0.087)** |  |  |  |
| Vegetarian | 132 | 5.0±1.4 | SoA |
| Vegan | 198 | 5.3±1.5 | SoA |

**SD, strongly disagree; D, disagree; SoD, somewhat disagree; Un, undecided; SoA, somewhat agree; A, agree; SA, strongly agree.**

**Table S7. Differences between study subjects according to Public Regard, as assessed on scales ranging from 1 to 7**

| **Item / Diet type (p-value)** | **N** | **Mean** | **Description** |
| --- | --- | --- | --- |
| People who follow my dietary pattern are judged negatively for their food choices. (p=0.278) |  |  |  |
| Vegetarian | 132 | 3.0±1.8 | SD |
| Vegan | 198 | 2.7±1.8 | SD |
| People who follow my dietary pattern tend to receive criticism for their food choices. (p=0.337) |  |  |  |
| Vegetarian | 132 | 1.9±1.2 | SD |
| Vegan | 198 | 2.1±1.5 | SD |
| Following my dietary pattern is associated with negative stereotypes. (p=0.877) |  |  |  |
| Vegetarian | 132 | 3.0±1.8 | SD |
| Vegan | 198 | 2.9±1.9 | D |
| **Public regard definition total. (p=0.833)** |  |  |  |
| Vegetarian | 132 | 2.6±1.3 | D |
| Vegan | 198 | 2.6±1.5 | D |

**SD, strongly disagree; D, disagree; SoD, somewhat disagree; Un, undecided; SoA, somewhat agree; A, agree; SA, strongly agree.**

**Table S8.** Differences between study subjects according to Strictness, as assessed on scales ranging from 1 to 7.

| **Item / Diet type (p-value)** | **N** | **Mean** | **Description** |
| --- | --- | --- | --- |
| I can be flexible and sometimes eat foods that go against my dietary pattern (p=0.493) |  |  |  |
| Vegetarian | 132 | 5.5±3.1 | A |
| Vegan | 198 | 5.3±1.9 | A |
| From time to time, I eat foods that go against my dietary pattern. (p=0.087) |  |  |  |
| Vegetarian | 132 | 5.5±1.8 | A |
| Vegan | 198 | 5.8±1.7 | A |
| I would eat a food product that goes against my dietary pattern if I were to hear that it tastes exceptionally good. (p=0.116) |  |  |  |
| Vegetarian | 132 | 5.8±1.7 | A |
| Vegan | 198 | 6.1±1.6 | A |
| **Strictness definition total. (p=0.425)** |  |  |  |
| Vegetarian | 132 | 5.6±1.8 | A |
| Vegan | 198 | 5.7±1.5 | A |

**SD, strongly disagree; D, disagree; SoD, somewhat disagree; Un, undecided; SoA, somewhat agree; A, agree; SA, strongly agree.**
